# The Ethics of Local Anæsthesia

**Published:** 1913-01-11

**Authors:** 


### PROBLEMS IN ADMINISTRATIVE MEDICINE.

(Carefully thought-out Contributions to this Section will be welcome.)

### The Ethics of Local Anæsthesia.

Since the introduction of cocaine as a local anæsthetic the medical profession has had plenty of opportunity to investigate the merits and demerits of the new method of inducing unconsciousness of physical pain in patients about to be operated upon. There are to-day so many local anæsthetics which clamour for recognition and popularity that it is obvious to every unprejudiced mind that local anæsthesia is steadily gaining ground. At the same time it is equally obvious that experience has shown that the various methods of local anæsthesia are not so simple and so utterly devoid of danger as enthusiastic advocates would have had us believe ten years ago. A careful record of fatalities—or of such at least as are available for reference in the records to be found in published papers—has proved that local anæsthesia may be as dangerous in its immediate results as general. Compared grossly with general anæsthesia, local anæsthesia is certainly found wanting; relatively, however, it has certain merits which every surgeon is prepared to consider carefully.

### Its Administrative Advantages.

While it is unnecessary here to enter into details (since such have more especially a scientific than an administrative value) it may briefly be stated that local anæsthesia offers the following advantages over general narcosis. It does away, to some extent, with the dangerous and not to be calculated factor of shock; it avoids largely the serious pulmonary complications which may, and often do, follow ether or chloroform anæsthesia; it obviates the resultant period of unconsciousness for the patient, a period which is administratively important since a nurse is needed to watch the patient when "coming round"; it does not make the patient unconscious, so that, in certain conditions, the operator has the advantage of being able to obtain the patient's consent to further measures; it does not prevent voluntary movement on the part of the patient; and finally, what is most important of all, it enables the surgeon to deal with a case almost singlehanded, whereas when general anæsthesia is used the services of an anæsthetist are required. All these advantages, it is easy to see, are also objections in some degree. The presumption that with local anæsthesia there is less likely to be shock is not always borne out by experience; with the older anæsthetics, stovaine and cocaine, shock was not infrequently an after result, although it is true that with more modern methods of "blocking" the larger nerve trunks this complication has been largely done away with. Similarly, while pulmonary complications are very rare when local anæsthesia is used, septic complications are not so rare, especially when spinal anæsthesia has been employed in a septic case. This is so well known that lumbar anæsthesia is regarded as contra-indicated in a case which is considered a septic one. It is not wholly true that with local anæsthesia a nurse is unnecessary to guard the patient immediately after the operation; where the operation is small such watching may be unnecessary, but in every major case the surgeon would safeguard his own and his patients' interests best if he followed the usual course and appointed a special nurse to watch the patient for an hour or so afterwards.

The argument that local anæsthesia produces no unconsciousness, and that, therefore, such anæsthesia is preferable to general anæsthesia, is one which the hospital worker should consider very carefully; it is with this aspect of the question that we wish to deal here. Before doing so, however, it is as well to admit that the final merit of local anæsthesia, the fact that it enables the surgeon to dispense with the services of an anæsthetist, is undoubtedly a very great one. It is just in emergency operations, where assistance is limited, that local anæsthesia proves its value. That it is valuable, very valuable, no experienced surgeon will attempt to deny. But its value lies largely in the fact that it is an emergency method, a method to be used in special cases under special circumstances. That being the case, it is as well that hospital workers should devote some attention to the manner in which the various methods by means of which it is administered are employed at their institutions, and to consider in some detail what may be called, not without justification, "the ethics of local anæsthesia."

The broad basis on which rests all our hospital treatment is humanity. The consideration of the patients' interests and feelings is a prime necessity. When such consideration is regarded as a secondary matter it is invariably the case that the institution as well as its inmates suffer from such disregard. No elaboration of this point is needed; we believe every supporter of our voluntary hospitals will accept it as axiomatic.

### An Unjustifiable Procedure.

That being the case, in what circumstances is a surgeon justified in using local anæsthesia? It may be argued that the matter is entirely a scientific question—a matter, indeed, of choice guided by clinical and surgical experience. That is true, but only to a certain extent. There are undoubtedly conditions—such as diabetic gangrene—where local anæsthesia will be preferable to general, and, indeed, the surgeon may point to several other kinds of cases in which the choice between the two methods admits of but little doubt. But it is a fact that in some institutions local anæsthesia is not employed with such discrimination. In one London hospital, for example, it has become fashionable to employ spinal anæsthesia in operating upon children. The method is advocated by the surgeons who employ it on various technical grounds, which seem to us to fail in justifying the procedure when we take into consideration the fact that the psychical condition of the sick child is hardly such as will bear the strain of an operation performed while the little patient is conscious and able to listen to what is said around him. In operating on a conscious patient, indeed, the surgeon has to be perpetually on his guard; his assistants and everyone in the room have to hold themselves sternly in check or run the risk of hurting the patient in a manner which is the reverse of gentle. Then, again, there is a certain amount of after-pain in every operation performed under local anæsthesia. However painless the actual operation may be, owing to the fact that the anæsthetic has dulled the nerves for the time being, with the return of the sensation to the dulled part, and the subsequent hyperæmia when adrenalin has been used, comes an aching pain that persists for some time, and that is necessarily badly borne by children. Of course, against such after-pain the surgeon can and does guard by administering anodynes such as morphia, omnopon, or scopolamine, or by injecting urea-quinine solutions. Similarly much may be done to guard against the nervousness and sensitiveness of the patient while on the operating table by a preliminary injection of an anodyne. The combination of omnopon with tropacocaine, or in a case of spinal anæsthesia, undoubtedly has that great advantage that it induces a period of lethargy during which the patient is in a semi-unconscious state which makes him but little responsive to outside psychical stimuli. But where such precautions are not used—and it is apparent that they cannot always be used—the patient who has to undergo a severe operation under local anæsthesia has to face an ordeal which may well frighten a sensitive person.

Thus discrimination in the use of local anæsthetics is indispensable, and from an administrative point of view it is well worth the trouble to insist that the surgical staff shall prepare a list of all operations done under local anæsthesia, stating the reasons why local was chosen in preference to general anæsthesia, the conditions of the operation, and the results to the patient. Such a return would at least enable the managers or the superintendent to find out to what an extent local anæsthesia is being used in the institution, and may be the means to ensure that where such methods are used the interests of the patient shall not be overlooked.

### The Committee's Relation to the Surgeon.

Such interest as a return would give in what goes on in the operating theatre of a hospital is a legitimate interest for the committee-man to take. It does not mean undue interference with the surgeons; it means merely a practical interest in the efficient working of the institution. When a visitor, lay or professional, coming unexpectedly upon a patient who has just undergone an operation under local anæsthesia and is being wheeled back into the ward, sees such a patient obviously the worse for the experience, the momentary impression is not easily effaced. That impression, generally, is one of disgust, for the mental and physical strain which the patient has undergone gives to his features an expression of poignant misery and suffering which must strike the humane observer. It is difficult then to judge of the rights or wrongs of the case; all that the visitor sees is a suffering individual, whose anguish is the direct result of a method which should be used in exceptional cases under exceptional conditions. Where that method is used the greatest pains should be taken to remove the patient with the least possible delay, and to get him back into a private room where he can have the assistance of a nurse. To make such a patient walk back to the ward is absolutely indefensible, and is a practice which, in English hospitals at least, should be severely condemned.

